# Supplementary material for: Phototherapeutic Induction of Immunogenic Cell Death and CD8+ T Cell-Granzyme B Mediated Cytolysis in Human Lung Cancer Cells and Organoids
Source: Cancers (Basel). 2022 Aug 25;14(17):4119. doi: 10.3390/cancers14174119 (PMC9454585; doi:10.3390/cancers14174119)
Supplement: Supplementary file 1 [file cancers-14-04119-s001.zip › cancers-1782902-supplementary.pdf]

cancers

MDPI

## Supplementary Materials

**Table S1**. Chemicals, enzymes and other reagents

| Name | Company | Cat. Number |
|---|---|---|
| Phenotiazonium salt, methylene blue | Sigma-Aldrich | M9140 |
| Cell lysis buffer (10x) | Cell Signaling Technology | 9803 |
| 4x Laemmli sample buffer | Bio-RAD | 161-0747 |
| Cell staining buffer | BioLegend | 420201 |
| Fixation buffer | BioLegend | 420801 |
| Collagnase | Merck/Sigma Al-drich | C9407-100 mg |
| DNase | Merck/Sigma Al-drich | DN25-100 mg |
| Red Blood cell lysis buffer | Roche | 4257400 |
| 0.05 % Trypsin-EDTA (1x) | Gibco | 25300-054 |
| Cultrex growth factor reduced BME type2 (Matrigel) | Biotechne UK | 3533-010-02 |
| Granzyme B inhibitor IV | Calbiochem | 36805 |
| WST1 assay kit | Sigma | 5015944001 |
| FITC Annexin V Apoptosis Detection | BioLegend | 640914 |
| Beads | Invitrogen | 01-1111-42 |

**Table S2**. Cell media and components

| Name | Company | Cat. Number |
|---|---|---|
| DMEM | Thermo Fisher Scientific | 21969-035 |
| DMEM-F12 (a phenol red-free medium) | Thermo Fisher Scientific | 2104025 |
| RPMI 1640 | Thermo Fisher Scientific | 21875034 |
| Advanced DMEM/F12 | Thermo Fisher Scientific | 12634028 |
| Foetal bovine serum | Thermo Fisher Scientific | 10500064 |
| Penicillin/Streptomycin | Thermo Fisher Scientific | 15140122 |
| L-Glutamine | Gibco | 25030-024 |
| Recombinant human IL-2 (rhIL-2) | Gibco | PHC0027 |
| LEAF anti-human CD3 antibody | BioLegend | 300314 |
| Ultra-LEAF anti-human CD28 antibody | BioLegend | 302934 |

**Table S3**. Antibodies for immunoblotting

| Antibody | Company | Cat. Number |
|---|---|---|
| Bcl2 | abcam | ab182858 |
| Calreticulin (D3E6) | Cell Signaling Technology | 12238 |
| β Actin | Cell Signaling Technology | 3700 |
| Rabbit IgG HRP linked | Cell Signalling Technology | 7074 |

| | | |
|---|---|---|
| Mouse IgG HRP linked | Cell Signaling Technology | 7076 |

**Table S4.** Antibodies r immunofluorescence

| Antibody | Company | Cat. Number |
|---|---|---|
| Calreticulin (D3E6) | Cell Signaling Technology | 12238 |
| Granzyme B (496B) | Thermo Fisher Scientific | 14-8889-82 |
| Pan-Cytokeratin (AE1/AE3) (PanCK) | abcam | ab27988 |
| Goat anti-Rabbit Ig (Alexa Fluor488) | Thermo Fisher Scientific | A-11012 |
| Donkey anti-Rat IgG H&L (Alexa Fluor647) | abcam | ab1501555 |
| Goat anti-Mouse Ig (Alexa Fluor594) | Thermo Fisher Scientific | A-11032 |

**Table S5.** Immunofluorescence probes and dye

| Probe/Dye | Company | Cat. Number |
|---|---|---|
| GrzB probe H5 | The University of Edinburgh | - |
| ActinGreen 488 ready probes | Thermo Fisher Scientific | R37110 |
| Toto™ 3-Iodide probe | Invitrogen | T3604 |
| Nuc Blue Fixed cells stain ready probe | Thermo Fisher Scientific | R37606 |
| CellEvent caspase3/7 Green Ready Probe Reagent | Thermo Fisher Scientific | R37111 |
| CellTracker™ Red CMTPX dye | Thermo Fisher Scientific | C34552 |
| Zombie-UV kit | BioLegend | 423101 |

**Table S6.** Antibodies for flow cytometry

| Antibody | Company | Cat. Number |
|---|---|---|
| PE anti-Calreticulin (EPR3924) | abcam | 205977 |
| FITC anti-HLA-ABC | BioLegend | 311404 |
| APC anti-CD54 (ICAM1) | BioLegend | 353112 |
| APC/Cy7 anti-CD45 | BioLegend | 368516 |
| FITC anti-CD326 (EpCAM) | BioLegend | 324204 |
| BV421 anti-CD8a | BioLegend | 301035 |
| FITC anti-CD8a | BioLegend | 301006 |
| FITC anti-Mouse IgG isotype control | BioLegend | 4002008 |
| PE anti-Rabbit IgG isotype control | abcam | 209478 |
| APC anti-Mouse IgG isotype control | BioLegend | 400120 |
| BV421 anti-Mouse IgG isotype control | BioLegend | 400157 |

**Table S7**. Human lung cancer organoid non complete media formulations

| ADF+++ media | Working Concentration | Company |
|---|---|---|
| Advanced DMEM/F12 | 1x | Life Technologies |

| | | |
|---|---|---|
| 1000x Glutamax | 1x | Life Technologies |
| 1M Hepes | 1mM | Life Technologies |
| Penicillin/Streptomycin | 100U/130μg per ml | Life Technologies |

**Table S8**. Human lung cancer organoid complete media formulations

| **Component** | **Signalling pathway** | | **Supplier** | **Catalogue number** | **Final concentration** |
|---|---|---|---|---|---|
| | **Activation** | *Blocking* | | | |
| R-Spondin 1 | **Wnt/β-Catenin signalling** | | Peprotech | 120-38 | 150 ng·ml$^{-1}$ |
| FGF 7 | **FGFR2b signalling** | | Peprotech | 100-19 | 25 ng·ml$^{-1}$ |
| FGF 10 | **FGFR2b signalling** | | Peprotech | 100-26 | 100 ng·ml$^{-1}$ |
| Noggin | | *TGF-β signalling* | Peprotech | 120-10C | 100 ng·ml$^{-1}$ |
| hEGF | **Growth, proliferation** | | R&D Systems | PHG0311 | 50 ng ml$^{-1}$ |
| CIR-99021 | **WNT signalling** | | Merck | SML1046-25mg | 3μM |
| A83-01 | | *TGF-β signalling* | Tocris | 2939 | 100 nM |
| Y-27632 | | *ROCK signalling* | Abmole | Y-27632 | 10 μM |
| SB202190 | | *p38 MAPK signalling* | Sigma | S7067 | 10 μM |
| B27 supplement | **Insulin signalling** | | Gibco | 17504-44 | 1x |
| N-Acetylcysteine | Antioxidant | | Sigma | A9165-5g | 1.25 mM |
| Nicotinamide | Co-enzyme precursor | | Sigma | N0636 | 10 mM |
| GlutaMax 100x | Nutrient | | Invitrogen | 12634-034 | 1x |
| Hepes | Buffer | | Invitrogen | 15630-056 | 10 mM |
| Penicillin/Streptomycin | Antibiotics | | Invitrogen | 15140-122 | 100 U·ml$^{-1}$ / 100 μg·ml$^{-1}$ |
| Primocin | Antibiotic | | Invitrogen | Ant-pm-1 | 50 μg·ml$^{-1}$ |
| Advanced DMEM/F12 | Base medium | | Invitrogen | 12634-034 | 1x |

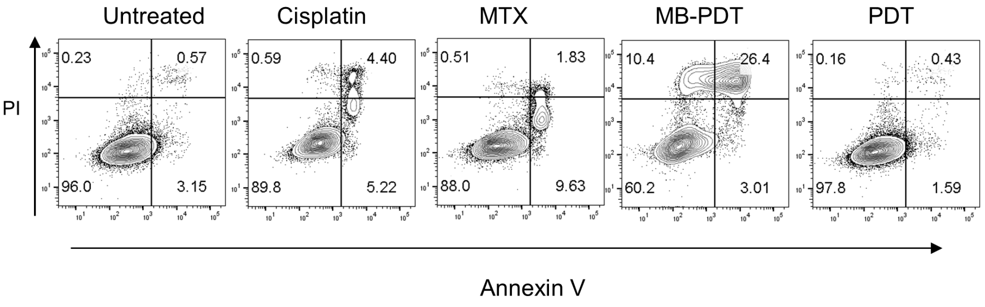


**Figure S1.** MB-PDT reduced A549 cell viability. Apoptosis demonstrated in A549 cells 24 hours post treatment by Annexin V assay. Representative histograms of flow cytometry showing PI (Y-axis) vs Annexin AF488 (X-axis) acquired on 5L LSR flow cytometry.

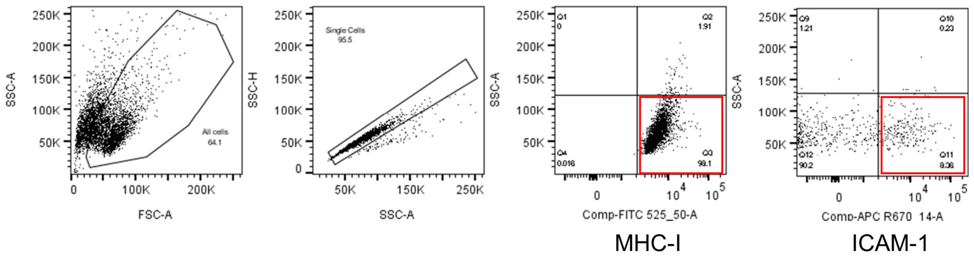


**Figure S2.** MB-PDT upregulated expression of MHC-I and ICAM-1, as markers of ICD. Gating strategy for flow cytometry of the expression of MHC-I and ICAM-1 in A549 cells 24h post-treatment. The expression of MHC-I and ICAM-1 was investigated in A549 cells 24 and 48 hours post-treatment by flow cytometry. Data were acquired on 5L LSR.

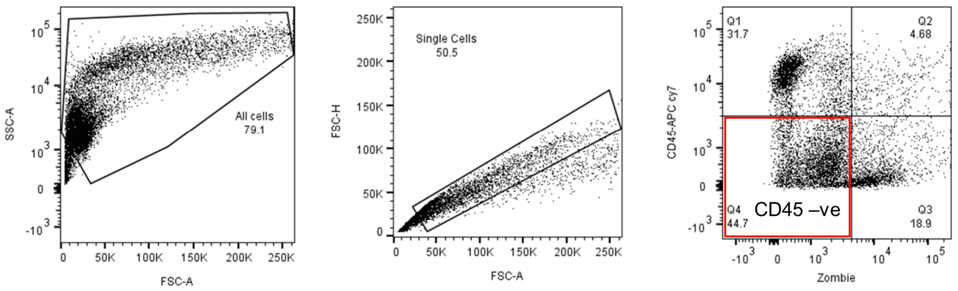


**Figure S3.** MB-PDT activated cytotoxic effect of CD8+ T cells. Gating strategy for flow cytometry of H1299 cells in co-culture with activated CD8+ T cells for 48 hours. Pre-gating was done on CD45-APC-cy7 cells and Zombie according to their light scatter characteristics using the 5L LSR flow cytometry.
